# Yi–Qi–Jian–Pi–Xiao–Yu–Xie–Zhuo Formula Improves Muscle Atrophy via Modulating the IGF-1/PI3K/Akt Signaling Pathway in 5/6 Nephrectomized Rats

**DOI:** 10.3389/fphar.2021.624303

**Published:** 2021-04-27

**Authors:** Hong Xia, Bingbing Zhang, Dan Yang, Chengyue Zhu, Jiudan Zhang, Hongbo Chen, Hongzhen Ma, Shouci Hu, Chao Xu, Chengqian Shi, Keda Lu, Peipei Zhang

**Affiliations:** ^1^ Department of Nephrology, The First Affiliated Hospital of Zhejiang Chinese Medical University, Hangzhou, China; ^2^ Postgraduate of Internal Medicine of Traditional Chinese Medicine, Zhejiang Chinese Medical University, Hangzhou, China; ^3^ Department of Orthopaedics, The Affiliated Guangxing Hospital of Zhejiang Chinese Medical University, Hangzhou, China; ^4^ Department of Endocrinology, The First Affiliated Hospital of Zhejiang Chinese Medical University, Hangzhou, China; ^5^ Department of Nephrology, The Second Affiliated Hospital of Zhejiang Chinese Medical University, Hangzhou, China

**Keywords:** Yi-Qi-Jian-Pi-Xiao-Yu-Xie-Zhuo formula, muscle atrophy, insulin-like growth factor l, phosphoinositide 3-kinase, akt

## Abstract

The Yi–Qi–Jian–Pi–Xiao–Yu–Xie–Zhuo (YQJPXYXZ) formula has been used for treating chronic kidney disease (CKD) for many years with good efficiency based on the cumulative empirical experience of previous practitioners. Impairment of the IGF-1/PI3K/Akt signaling pathway plays an important role in mediating muscle wasting. This study aimed to observe effects of the YQJPXYXZ formula on muscle atrophy in CKD rats and investigate its possible mechanism on regulation of the IGF-1/PI3K/Akt signaling pathway. The 5/6 nephrectomized rats were randomly allocated into 3 groups: the CKD group, the KT (compound α-ketoacid tablets) group, and the YQJPXYXZ group. Besides, sham-operated rats were included as the sham group. All rats were treated for 12 weeks. Results showed that administration of the YQJPXYXZ formula prevented body weight loss and muscle fiber size decrease. Moreover, the YQJPXYXZ formula increased the IGF-1 level of serum and skeletal muscle in CKD rats and enhanced the phosphorylation level of Akt. Furthermore, the YQJPXYXZ formula decreased the Atrogin1 and MuRF1 mRNA and MuRF1 proteins. In conclusion, our data demonstrated that the YQJPXYXZ formula improves muscle wasting in CKD rats, which might be associated with the modulation of the IGF-1/PI3K/Akt signaling pathway and inhibition of the ubiquitin–proteasome system (UPS).

## Introduction

CKD is characterized by progressive decline in renal function over months or years and is an increasing public health issue (Webster et al., 2017). Protein energy wasting (PEW) refers to loss of body protein mass and fuel reserves (Fouque et al., 2008). Surveys show that PEW is present in 18–75% of CKD patients undergoing maintenance dialysis therapy (Mehrotra and Kopple, 2001; Kalantar-Zadeh et al., 2003). The diagnosis of PEW mainly includes four aspects as follows: biochemical criteria; low body weight, reduced total body fat, or weight loss; a decrease in muscle mass; and low protein or energy intakes (Fouque et al., 2008). Muscle wasting seems to be the strongest predictor for the diagnosis of PEW in CKD (Kaysen, 2005; Fouque et al., 2008).

Many factors result in or accelerate muscle wasting in kidney disease. These include inflammation, acidosis (Kalantar-Zadeh et al., 2004), hemodialysis (HD) treatment (Kaplan et al., 1995), hyperglucagonemia (Sherwin et al., 1976), hyperparathyroidism (Kopple et al., 1980), endocrine disorders such as resistance to insulin (Mak, 1996) and insulin-like growth factor-1(IGF-1) (Ding et al., 1996), and so on. IGF-1 is a kind of protein that promotes anabolism, and its inhibition plays an important role in muscle atrophy in end stage renal disease (ESRD) (Kopple et al., 2007). The IGF-1/PI3K/Akt pathway promoting muscle hypertrophy prevents expression of muscle atrophy–induced UPS, namely, the muscle-specific ubiquitin ligases Atrogin1 and MuRF1 (Stitt et al., 2004).

PEW directly correlates with mortality and morbidity in patients with CKD (Kalantar-Zadeh et al., 2011). However, there have not been effective preventive and therapeutic interventions that delay muscle wasting so far. In recent years more and more traditional Chinese medicine (TCM) formulas are used in patients with CKD in China and other Asian countries because of their characteristics, such as less adverse effect, abundant resources, low cost, and stable effect (Zhong et al., 2013; Zhong et al., 2015). The YQJPXYXZ formula, modified from the 'Buyang Huanwu Decoction' which was first recorded in <Correction in the Errors of Medical Works>, is significant in replenishing qi, invigorating the spleen, eliminating stasis, and purging dampness turbidity. It is formulated with nine herbs, including Radix Astragali, Radix Cyathulae, Semen Persicae, Lumbricus, Chinese rhubarb, plantain herb, Radix Codonopsis, Poria, and white Atractylodes rhizome. According to yin–yang and the five elements theory of TCM, the YQJPXYXZ formula is in a weight ratio of 30:12:12:12:10:20:15:15:15. The YQJPXYXZ formula has been widely used in treating CKD with good efficiency for many years (Lu et al., 2014; Lu et al., 2016). It has been a standard hospital prescription at the First Affiliated Hospital of Zhejiang Chinese Medical University (Hangzhou, China). Despite the good efficiency, the underlying molecular mechanism and pharmacological action of the YQJPXYXZ formula remain unclear. In this study, we examined whether the YQJPXYXZ formula would delay muscle atrophy in 5/6 nephrectomized rats by modulating the IGF-1/PI3K/Akt signaling pathway.

## Materials and Methods

### TCM Preparation: Yi–Qi–Jian–Pi–Xiao–Yu–Xie–Zhuo

The nine herbs are Astragali Radix (30 g), Radix Cyathulae (12 g), Semen Persicae (12 g), Lumbricus (12 g), Chinese rhubarb (10 g), plantain herb(20 g), Radix Codonopsis (15 g), Poria (15 g), and white Atractylodes rhizome (15 g). The nine herbs were purchased from Hangzhou Huadong Pharmaceutical Co., Ltd. (Hangzhou, China). The plant materials were authenticated by Dr. Xishan Xu based on their morphological characteristics. The voucher specimens were kept at the Pharmaceutical Department, the First Affiliated Hospital of Zhejiang Chinese Medical University with numbers 200414, 200406, 200420, 200406, 200514, 200511, 200225, 200306, and 200523, respectively. Assurance of quality control for all the materials was validated according to the 2015 edition of Pharmacopoeia of the people’s Republic of China.

Nine herbal ingredients were mixed by proportions which are shown as numbers that are within the brackets following each scientific name of an herb. The mixture was extracted sequentially with 0.6 L boiling water twice for 1 h. The extracted liquid was mixed and filtered. After filtration, the dregs of the formula were removed. The filtered liquid was lyophilized and then crushed into a thin powder. The powder was suspended in distilled water to a fixed concentration (1.47 g/ml, 6.25 is the conversion coefficient on the basis of body surface area between human and rat) and stored at 4°C. The YQJPXYXZ formula was subsequently used for all experiments in this research. Some powder was stored in an –80°C refrigerator before injection into an HPLC system for analysis.

### HPLC Analysis

Standards of calycosin-7-*O*-*β*-D-glucoside, astragaloside IV, aloe emodin, atracylenolide III, emodin, chrysophanol, and physcion were purchased from the China Institute of Food and Drug Verification and Research (Beijing, China). Standards of amygdalin, lobetyolin, calycosin, astragaloside III, and formononetin were purchased from Sichuan Victory Biological Technology Co., Ltd. (Chengdu, China). Chromatographic grade acetonitrile was purchased from Merck (Darmstadt, Germany). All the chemical reagents used in this research were of analytic grade.

The YQJPXYXZ powder was subjected to HPLC analysis. 1.0 g YQJPXYXZ powder sample was accurately weighed and sonicated in 20 ml of 75% methanol by ultrasonic extraction for 30 min at 25°C. The weight loss was compensated by adding 75% methanol after extraction. Then the solution was centrifuged at 4,000 rpm for 5 min (LD5-2 A Low-speed centrifuge, Jingli, Beijing, China), and the supernatant was filtered through a membrane with 0.22 µm pores for analysis. All solutions were stored at 4°C until use. HPLC-QQQ-MS/MS analysis was done using a Shimadzu LCMS 8045 instrument coupled with electron spray ionization (Shimazdu, Kyoto, Japan). Chromatographic separation was accomplished on a Thermo Scientific Hypersil GOLD column (Shimazdu, Kyoto, Japan). Chromatographic separation was accomplished on a Thermo Scientific Hypersil GOLD column (150 × 4.6 mm, 3 μm, Thermo Fisher Scientific, Massachusetts, United States) at a flow rate of 0.3 ml/min and an injection volume of 5 μL. The mobile phase was composed of acetonitrile (A) and water (B) with the following gradient elution program: 0–45 min, 22%–95% A; 45–52 min, 95% A. The solution was injected into the HPLC system (Shimadzu, Kyoto, Japan) for analysis in triplicate.

### CKD Model Set-Up

The experimental and feeding protocols were in accordance with National Health guidelines and were approved by the First Affiliated Hospital of Zhejiang Chinese Medical University Institutional Animal Care and Use Committee. Male Sprague–Dawley rats were purchased from the Zhejiang Chinese Medical University Laboratory Animal Research Center, certification no. SYXK (ZHE) 2018–0012, weighing 130–150 g. The animals were kept in the animal laboratory in a controlled environment, a cycle of 12 hours of darkness–light and temperature 22 ± 1°C, with free access to food and water. The animals adapted to their surroundings for 1 week before the experiments were started. Male rats were randomly assigned to either the 5/6 nephrectomized group or the sham-operated group. Each animal in the nephrectomized group underwent a 5/6 nephrectomy, consisting of removing the upper and lower one-third parts of the left kidney, and a right unilateral nephrectomy after 2 weeks. In the sham group, anesthesia and surgery were performed without removal of the kidney mass. The rats were given a daily low protein diet the day after the operation. Then, 4 weeks after the operation, the 5/6 nephrectomy group was randomly separated and maintained as three different groups: the CKD group, the KA group, and the YQJPXYXZ group. The sham group acted as the control. Each group included eight rats.

### Experimental Diets

The low protein diet was provided by Jiangsu Synergetic Pharmaceutical Bioengineering Company Limited (production license no: Susi Certificate (2014) 01,008) and given to the rats the day after the operation. The low protein diet was as follows (g/Kg): casein 60, starch 539, gelatinized starch 130.5, sucrose 100, soybean oil 70, microcrystalline cellulose 50, mineral salt mixture 35, vitamin mixture 10, L-cystine 3.0, and choline chloride 2.5. KT was provided by Beijing Fresenius Kabi Pharmaceutical Company Limited. The KT composition was as follows (mg/630 mg): racemic keto isoleucine, 67 mg; ketones leucine, 101 mg; phenylalanine ketone, 68 mg; ketones valine, 86 mg; dl-methionine hydroxy, 59 mg; lysine acetate, 105 mg; threonine, 53 mg; tryptophan, 23 mg; histidine, 38 mg; tyrosine, 30 mg; and total N, 36 mg. The concentration of KT suspension was 78.75 mg/ml, and it was stored at 4°C. The sham group and the CKD group were administered with physiological saline. The KT group was administered with KT suspension, and the YQJPXYXZ group was administered with the YQJPXYXZ formula. The feeding volume was 1 ml/100 g once a day. These administrations were given to the groups for a period of 12 weeks. The method involves orally administering to the rat through a feeding needle and swallowing. The rats in all groups had free access to the low protein diet, and water was provided ad libitum.

### Renoprotective Effect of YQJPXYXZ Formula on the Remnant Kidney

After 12 weeks of treatment, the rats were terminated, and the remnant kidney tissues were fixed in paraformaldehyde and embedded in paraffin. The tissues were sectioned and stained with hematoxylin and eosin (H&E) staining.

### Biochemical Analysis

After 12 weeks of treatment, the rats were terminated using sodium pentobarbital, and blood samples were subsequently collected. Serum creatinine (Scr), blood urea nitrogen (BUN), serum albumin (ALB), and urine creatinine (Ucr) were measured using DiaSys Diagnostics Systems GmbH following the manufacturer’s instructions.

### Testing of 24 h Urine Protein

24 h urine samples were collected by using metabolism cages. The 24 h urinary protein excretion was measured with DiaSys Diagnostics Systems GmbH following the manufacturer’s instructions.

### Muscle Histology and Myofiber Cross-Sectional Area Measurements

Tibias anterior (TA) muscle samples were fixed in paraformaldehyde and embedded in paraffin. The muscles were sectioned and stained with hematoxylin and eosin (H&E) in line with the standards. In each muscle, six sections of fifty contiguous myofibres were demarcated so that an average of 200 fibers was obtained for fiber area measurement in each group. The image morphometry program was Image Pro Plus6.0 software (Media Cybernetics, Bethesda, MD, United States).

### IGF-1 Concentration Test

The concentration of IGF-1 in serum was measured using the appropriate ELISA kit (R&D Systems, Inc., Quantikine ELISA SMG100) according to the manufacturer’s instructions.

### Quantitative Real-Time PCR

Total RNA was isolated from the anterior tibial muscle using Trizol (Invitrogen, Carlsbad, CA, United States). RNA concentration and integrity were assessed. cDNA was synthesized using a TaKaRa (RR037A) PrimeScript™ RT reagent Kit at 30°C for 10 min, followed by incubation at 42°C for 60 min and at 95°C for 5 min. The genes analyzed were IGF-1, Atrogin1, MuRF1, and β-actin (reference gene) (Table 1). All primers were synthesized by Invitrogen. Quantitative real-time PCR was run for all genes separately, and amplifications were performed by the ABI Prism 7900HT Sequence Detection System (Applied Biosystems) using TaKaRa (RR820A) TB Green®Premix Ex Taq™ II. The results were quantified as Ct values, where Ct is defined as the threshold cycle of the polymerase chain reaction at which the amplified product is first detected. The expression was normalized by β-actin levels as an endogenous reference.

**TABLE 1:** Primer sequences.

Gene	Forward	Reverse
IGF-1	5′-TAC​TTC​AAC​AAG​CCC​ACA​GG-3′	5′-ACA​TCT​CCA​GCC​TCC​TCA​GA-3′
Atrogin1	5′-CCA​CTC​TAC​ACT​GGC​AAC​AGC​AG-3′	5′-AGG​CAG​GTC​GGT​GAT​CGT​GAG-3′
MuRF1	5′-CCT​CGT​GCC​GCC​ATG​AAG​TG-3′	5′-GTC​GAT​GAT​GTT​CTC​CAC​CAG​CAG-3′
β-Actin	5′-GGA​GAT​TAC​TGC​CCT​GGC​TCC​TA-3′	5′-GAC​TCA​TCG​TAC​TCC​TGC​TTG​CTG-3′

### Western Blot Analysis of Akt and p-Akt Expression in Rat Gastrocnemius Muscle

Gastrocnemius muscles were lyzed using a Triton X-100-based lysis buffer that contained 1% Triton X-100, 150 mM NaCl, 10 mM Tris (pH 7.5), 5 mM EDTA, 5 mM NaN3, 10 mM NaF, and 10 mM sodium pyrophosphate. Muscle extracts were separated using SDS-PAGE and then transferred to a PVDF membrane (Millipore Corporation, Billerica, MA, United States). After blocking, the blots were developed using rabbit monoclonal anti-pAkt antibody or rabbit monoclonal anti-Akt antibody. The blots were then hybridized using HRP-conjugated goat anti-rabbit IgG (Abcam, United States) and developed with a chemiluminescence kit (Life Sciences, Inc., United States). The western band density that corresponded to the Akt or p-Akt or GAPDH was determined using an image analysis system. The detected density was the representation of the expression level of each protein. The density of p-Akt was calculated versus the density of Akt, and the result was shown as the proportion. The proportion was plotted as a bar graph with the value of group N set to be 1. Single antibodies: anti-pAkt (CST, INC. 8200s); anti-Akt (CST, INC. 8200s); anti-MuRF1 (SANTA CRUZ, INC. sc-398608); and anti-IGF-1 (Abcam, ab106836).

### Statistical Analysis

Data were analyzed with SPSS 13.0 (SPSS Inc: Chicago, IL, United States). The results are shown as mean ± SD. Statistical significance between groups of data was analyzed by the nonpaired Student’s t test. Evaluation of statistical significance among several groups was carried out by using ANOVA. Statistical significance was taken as *p* < 0.05.

## Results

### HPLC of YQJPXYXZ

**FIGURE 1:**
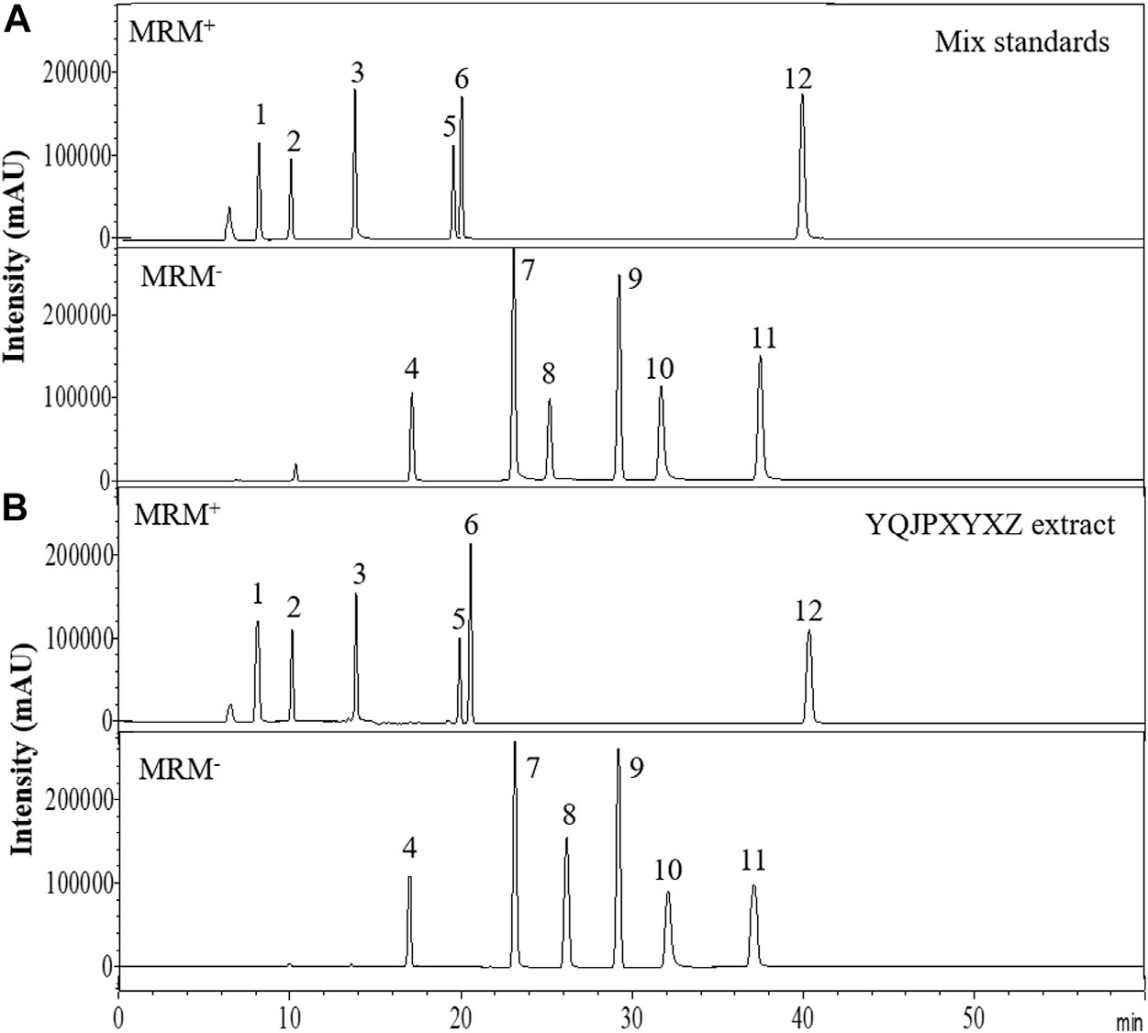
HPLC analysis of the YQJPXYXZ formula **(A)**. Mix standards **(B)**. YQJPXYXZ formula extract: (1) amygdalin; (2) calycosin-7-O-*β*-D-glucoside; (3) lobetyolin; (4) calycosin; (5) astragaloside IV; (6) astragaloside III; (7) formononetin; (8) aloe emodin; (9) atracylenolide III; (10) emodin; (11) chrysophanol; and (12) physcion.

**FIGURE 2:**
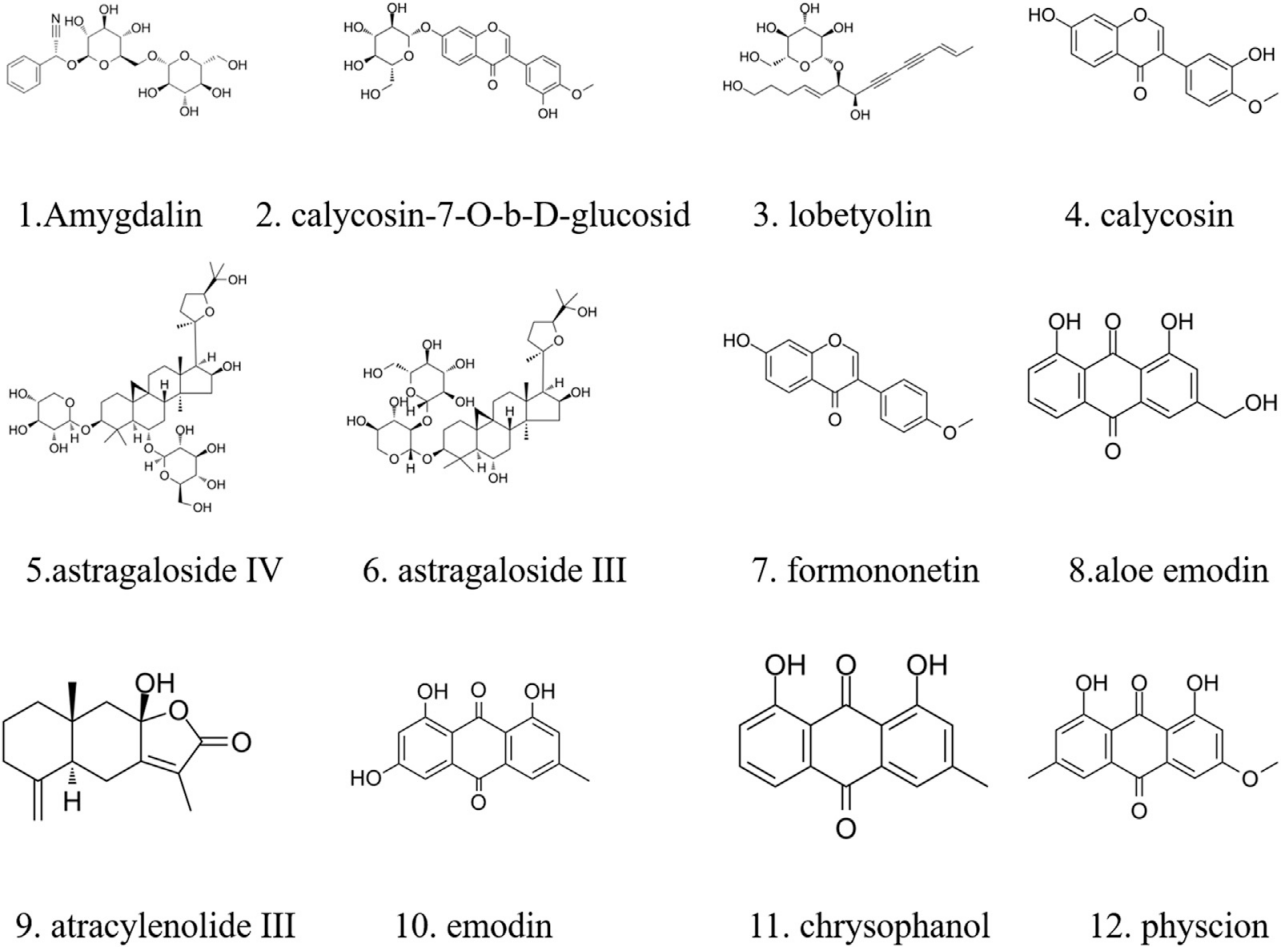
Chemical structure of the main active ingredients of the YQJPXYXZ formula.

The major components of YQJPXYXZ were analyzed by using the HPLC-QQQ-MS/MS method. By comparison with the standard reference compounds, twelve compounds were identified: 1) amygdalin, 2) calycosin-7-*O*-*β*-D-glucoside, 3) lobetyolin, 4) calycosin, 5) astragaloside IV, 6) astragaloside III, 7) formononetin, 8) aloe emodin, 9) atracylenolide III, 10) emodin, 11) chrysophanol, and 12) physcion. The percentage content of the twelve compounds was estimated using a calibration curve method. The minimal requirement for the amounts of astragaloside IV, chrysophanol, and physcion should be no less than 0.0031 mg/g, 0.0097 mg/g, and 0.0133 mg/g of the dried extract. The extract being used here met the aforementioned requirements. The concentration and retention time of the compounds are shown in Table 2. A representative chromatogram of YQJPXYXZ is shown in Figure 1. The chemical structure of the main active ingredients of the YQJPXYXZ formula is shown in Figure 2.

**TABLE 2:** Herbal concentration and retention time of 12 components in YQJPXYXZ.

No.	Compounds	Content (mg/g)	Retention time (min)
1	Amygdalin	0.0166	8.226
2	Calycosin-7-*O*-*β*-D-glucoside	0.0900	10.093
3	Lobetyolin	0.0147	13.719
4	Calycosin	0.1021	16.881
5	Astragaloside IV	0.0043	19.531
6	Astragaloside III	0.0218	20.129
7	Formononetin	0.0543	22.875
8	Aloe emodin	0.0677	24.955
9	Atracylenolide III	0.0288	29.142
10	Emodin	0.0443	31.631
11	Chrysophanol	0.0118	37.377
12	Physcion	0.014	39.834

### Renoprotective Effect of the YQJPXYXZ Formula on the Remnant Kidney

**FIGURE 3:**
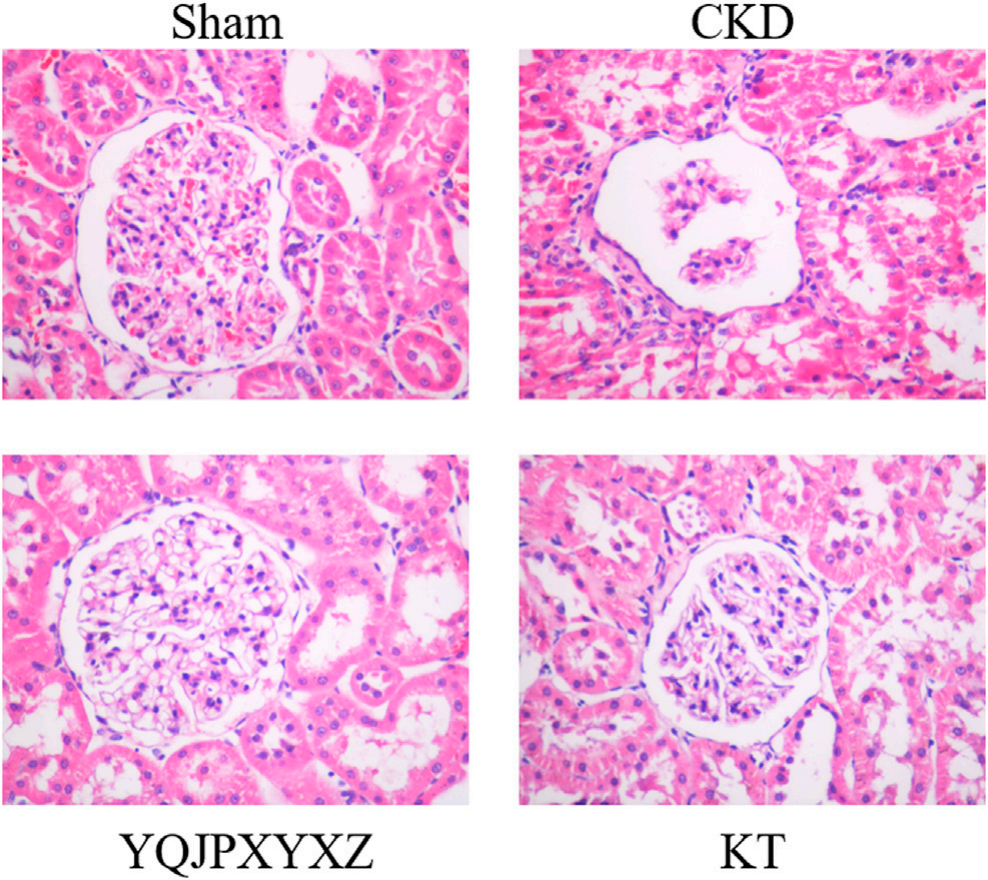
Renoprotective effect of the YQJPXYXZ formula on the remnant kidney. HE staining of the remnant kidney tissue. Magnification ×400.

Glomerulosclerosis and tubulointerstitial fibrosis were a prominent feature in the CKD group. These changes were attenuated by treatment with the YQJPXYXZ formula in the YQJPXYXZ group (Figure 3).

### Changes in Renal Function./YQJPXYXZ Improved Kidney Function and Serum ALB Level in 5/6 Nephrectomized Rats

Before administration, the level of Scr, BUN, and ALB did not differ significantly in the 5/6 nephrectomy groups. Compared with the sham rats, Scr, BUN, and ALB increased significantly in the 5/6 nephrectomized rats. After 12 °weeks of the treatments, the 5/6 nephrectomy groups displayed significantly higher Scr and BUN levels than the sham group. In the 5/6 nephrectomy groups, the YQJPXYXZ formula significantly decreased Scr and BUN levels, while KT significantly decreased Scr and tended to decrease BUN compared with the CKD group. Interestingly, the YQJPXYXZ formula was found to reduce the levels of Scr and BUN compared with the KT group. On the other hand, the ALB level was lower in the 5/6 nephrectomy groups than in the sham group. Among the 5/6 nephrectomy groups, the CKD group had a lower serum ALB level than the YQJPXYXZ and KT groups, but no statistical difference was observed between the YQJPXYXZ and KT groups (Table 3).

**TABLE 3:** Renal function data (means ± SD).

Group	Scr (umol/L)		BUN (mmol/L)		ALB (g/L)	
	0 weeks	12 weeks	0 weeks	12 weeks	0 weeks	12 weeks
Sham	28.00 ± 2.46	30.70 ± 1.77	2.70 ± 0.20	3.86 ± 1.66	31.93 ± 0.65	33.16 ± 0.69
CKD	65.43 ± 12.93^**^	63.80 ± 10.71^**^	9.40 ± 2.68^**^	11.41 ± 2.24^**^	29.58 ± 1.40^*^	24.07 ± 1.26^**^
YQJPXYXZ	61.69 ± 9.15^**^	43.15 ± 4.13^**#●^	9.37 ± 1.65^**^	7.54 ± 1.07^**#●^	30.21 ± 1.01^*^	25.98 ± 1.48^**△^
KA	62.35 ± 7.57^**^	50.35 ± 4.71^**△^	9.97 ± 2.00^**^	9.83 ± 1.83^**^	29.89 ± 1.44^**^	25.29 ± 0.93^**△^

*p < 0.05 versus sham, **p < 0.01 versus sham, △*p* < 0.05 versus CKD, ^#^ *p* < 0.01 versus CKD, ^●^ *p* < 0.05 versus KA.

### General Biochemical Parameters of Urine

Before the administration, the 24 h urine volume significantly increased in the 5/6 nephrectomy groups compared with the sham group. After 12 °weeks of the administration, the 24 h urine volume tended to decrease in YQJPXYXZ rats, but this decrease did not reach statistical significance. Before administration and after 12 °weeks of the administration, the level of 24 h urine protein in the 5/6 nephrectomy groups significantly increased compared with the sham group. As expected, 24 h urine protein after 12°weeks of the administration was decreased by YQJPXYXZ or KT treatment (Table 4).

**TABLE 4:** The 24 h urine volume and 24 h urine protein (means ± SD).

Group	24 h urine volume (ml)		24 h urine protein (mg)	
	0 weeks	12 weeks	Group	0 weeks
Sham	3.50 ± 0.71	14.00 ± 8.49	22.67 ± 2.51	21.03 ± 3.58
CKD	9.38 ± 2.29**	27.00 ± 9.06	37.00 ± 5.29^**^	34.50 ± 3.17^**^
YQJPXYXZ	9.20 ± 3.35**	24.75 ± 5.85	33.33 ± 1.53^*^	27.51 ± 0.58^*△^
KA	9.40 ± 3.58**	33.20 ± 23.77	34.33 ± 5.13^**^	28.88 ± 2.31^**△^

*p < 0.050.05 versus sham, **p < 0.01 versus sham, △*p* < 0.05 versus CKD.

### Effects of YQJPXYXZ on Muscle Fiber Cross-Sectional Area

**FIGURE 4:**
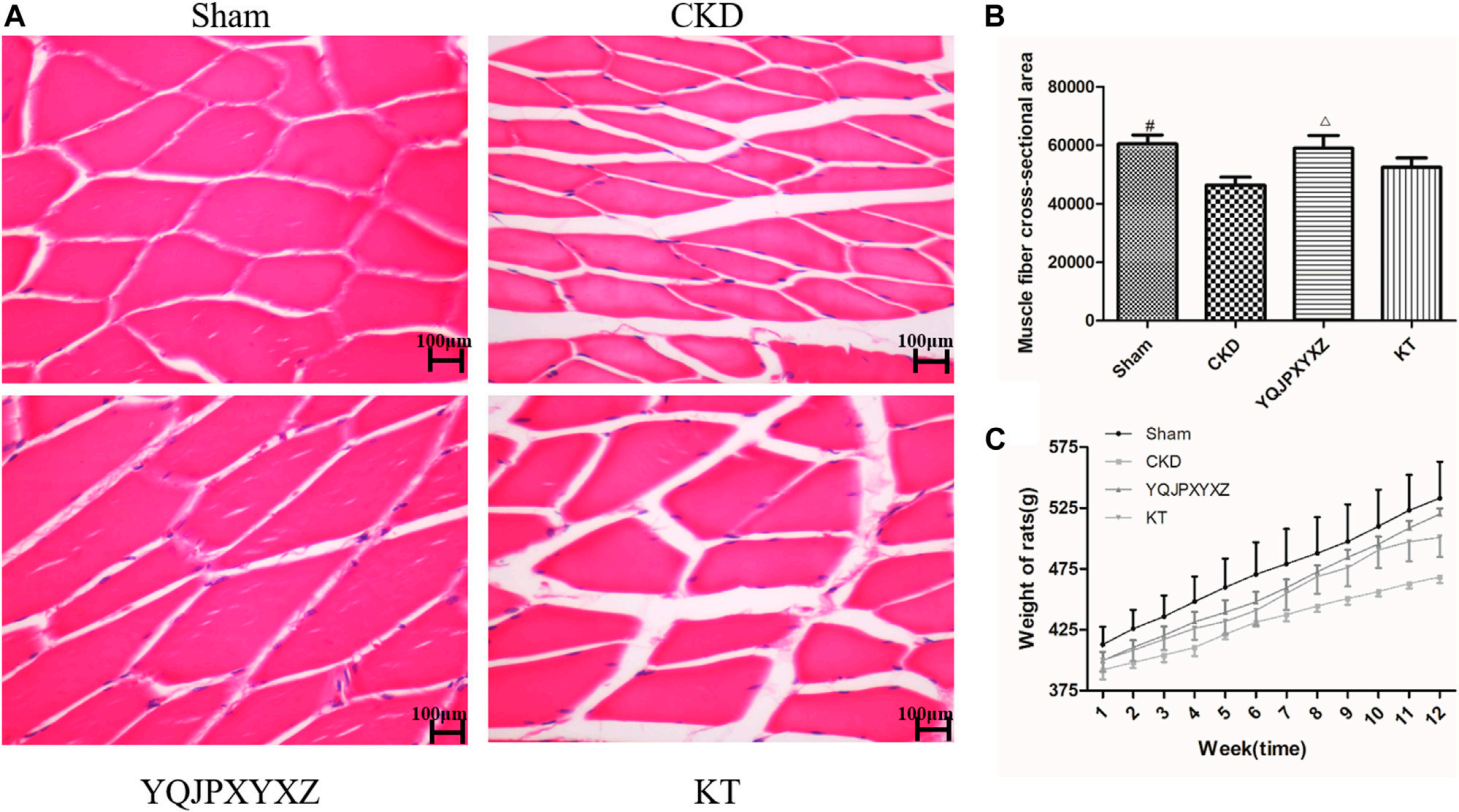
Effect of the YQJPXYXZ formula on muscle fiber cross-sectional area and body weight in 5/6 nephrectomized rats. **(A)** HE staining of the TA muscle. Scale bar = 100 g m. **(B)** Average fiber size of the HE-stained TA muscle. **(C)** Body weight. Results are presented as mean ± SD, *n* = 8 per group, ^#^ *p* < 0.01 versus CKD, and Δ*p* < 0.05 versus CKD.

We used the cross-sectional area of muscle fiber to evaluate muscle atrophy as previously described. The improved muscle mass was confirmed by an increase in the average cross-sectional area of myofibers in TA muscles in the YQJPXYXZ group. Representative views are shown in Figures 4A,B. The mean cross-sectional area of TA muscle in the CKD group was significantly lower than that in the sham group (P< 0.01). Compared with the CKD group, fiber atrophy was attenuated in the YQJPXYXZ group (*p* < 0.05).

### YQJPXYXZ Formula Increases Body Weight of CKD Rats

The body weight of CKD rats was significantly lower than that of sham rats before the treatment, but there were no differences in body weight among the 5/6 nephrectomy groups. Interestingly, we found that both YQJPXYXZ and KT tended to increase the body weight of rats. Moreover, the YQJPXYXZ group showed obvious improvement of body weight in the treatment of 11 and 12°weeks when compared with the CKD group (Figure 4C). Body weight did not significantly differ between the YQJPXYXZ group and the KT group.

### YQJPXYXZ Formula Increases Serum IGF-1 and Skeletal Muscle IGF-1 mRNA in CRF Rats

**FIGURE 5:**
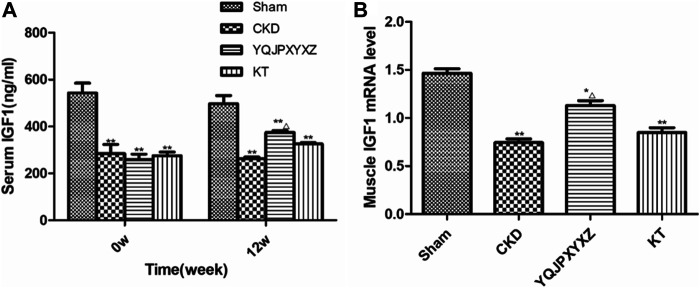
YQJPXYXZ formula increases Sentm IGF-I and skeletal muscle IGF-I mRNA in 5/6 nephrectomized rats. Results are presented as mean ± SD, *n* = 8 per group, *p< 0.05 versus sham, **p < 0.01 versus sham, and Δ*p* <0.05 versus CKD.

Before the treatment and after 12°weeks of the administration, the 5/6 nephrectomy groups exhibited a significant decrease of serum IGF-1 level compared to the sham group; however, YQJPXYXZ supplementation tended to increase the level of serum IGF-1, and the difference was statistically significant (Figure 5A).

After 12 weeks of the administration, mRNA expression of skeletal muscle IGF-1 was analyzed. As shown in Figure 5B, compared with the sham rats, muscle IGF-1 mRNA reduced in the 5/6 nephrectomized rats, interestingly, which was inhibited by YQJPXYXZ; KT supplementation tended to increase the level of skeletal muscle IGF-1 mRNA, but no statistical difference was observed compared with the CKD and YQJPXYXZ groups.

### YQJPXYXZ Formula Increases Skeletal Muscle IGF-1 and p-Akt in CRF Rats by Western Blot

**FIGURE 6:**
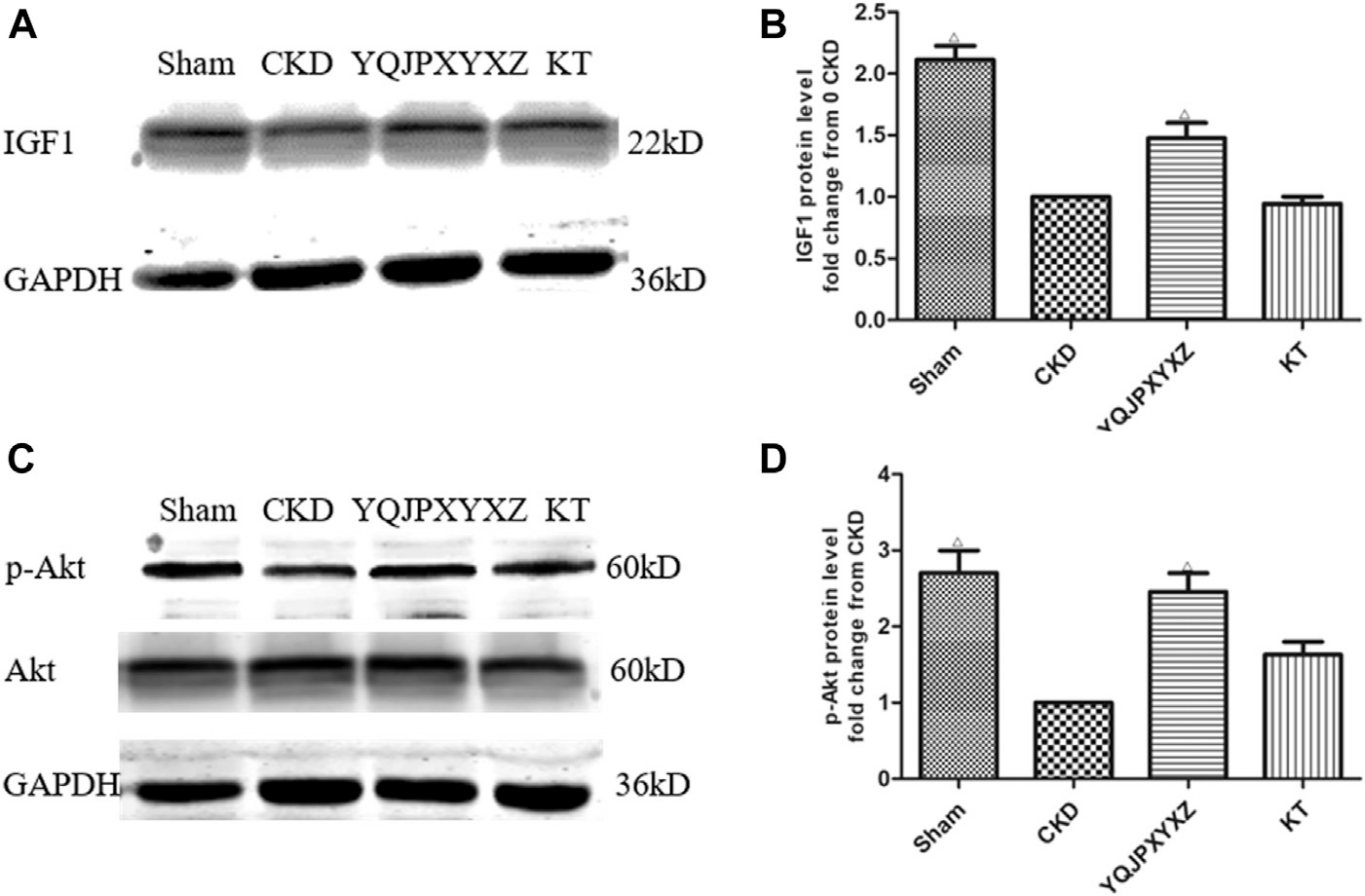
YQJPXYXZ formula increases skeletal muscle IGF-I and p-Akt in 5/6 nephrectomized rats by Western blot. Results are presented as mean ± SD, *n* = 8 per group, and Δ*p*<0.05 versus CKD.

We measured the expression of IGF-1 and p-Akt protein in CKD rats because others (Stitt et al., 2004) have shown that the IGF-1/PI3K/Akt pathway would suppress muscle wasting, and its suppression plays an important role in ESRD-induced muscle atrophy. We found that CKD caused a marked reduction of IGF-1 and p-Akt protein when compared with the sham group (Figure 6). However, the YQJPXYXZ formula increased that significantly compared with the CKD group. The expressions of IGF-1 and p-Akt protein in the KT group were not different from those in the CKD group.

### YQJPXYXZ Formula Inhibits UPS

**FIGURE 7:**
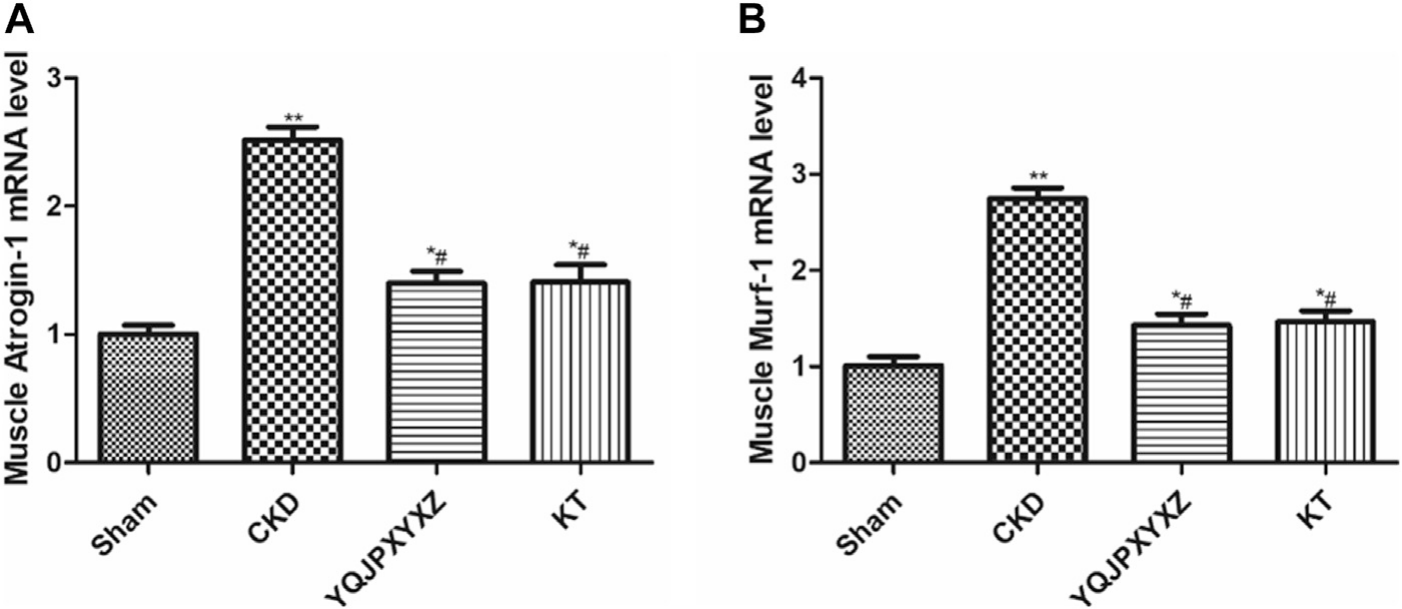
YQJPXYXZ formula inhibits the UPS in 5/6 nephrectomized rats. Results are presented as mean ± SD, n = 8 per group, *p< 0.05 versus sham, **p < 0.01 vs. sham, and ^#^ *p* < 0.01 versus CKD.

UPS is one of the major pathways involved in regulation of muscle wasting. Low p-AKA activity in muscle is associated with an increase in Atrogin1 and MuRF1 expression and protein degradation (Lee et al., 2004; Sandri et al., 2004). To further analyze whether there is an effect of the YQJPXYXZ formula on this pathway to regulate muscle atrophy, we evaluated the expression of Atrogin1 and MuRF1 mRNA. The CKD group displayed a significant increase in the mRNA expression of Atrogin1 and MuRF1, and the changes were inhibited by the YQJPXYXZ formula or KT administration (Figure 7).

### YQJPXYXZ Formula Reduces Skeletal Muscle Murf1 in CRF Rats

**FIGURE 8:**
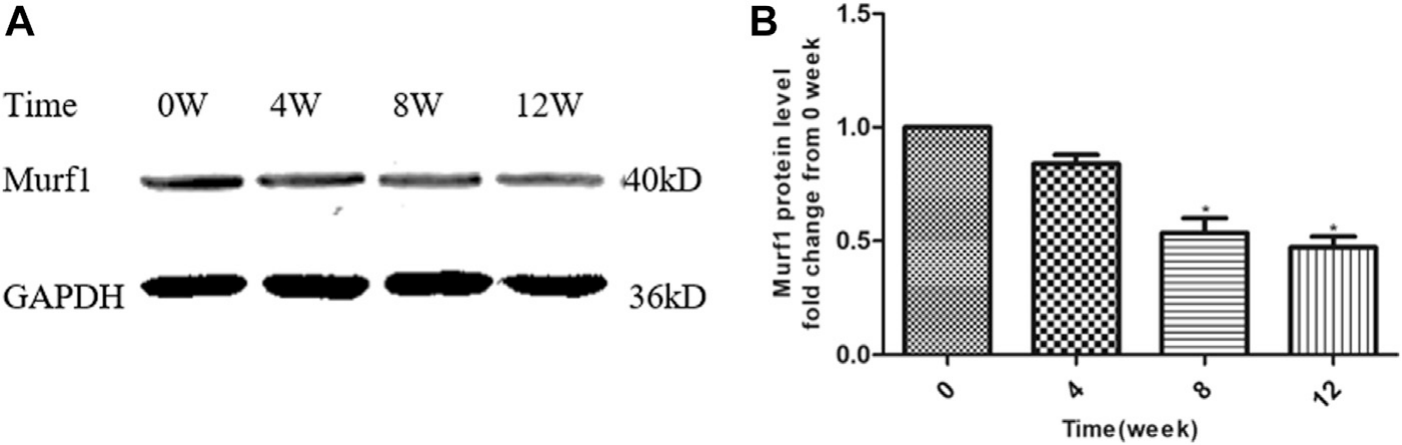
YQJPXYXZ formula reduces skeletal muscle Murf1 in 5/6 nephrectomized rats. Results are presented as mean ± SD, n = 8 per group, and *p < 0.05 versus 0 weeks.

At the end of this study, the Murf1 protein levels at different time points of YQJPXYXZ formula administration were examined. As Figure 8 shows, the YQJPXYXZ formula decreased the expression of the Murf1 protein level in a time-dependent manner. The Murf1 protein was significantly lower in weeks 8 and 12, respectively, than that before the YQJPXYXZ formula administration.

## Discussion

PEW characterized with muscle wasting is a serious complication of CKD and strongly associated with increased morbidity and mortality of patients (Caetano et al., 2016). Muscle wasting, that is, muscle atrophy, is well described in CKD patients, especially in incident and prevalent dialysis patients (Guarnieri et al., 1983; Carrero et al., 2008). Rat models with chronic renal failure display enhanced protein degradation and impaired protein synthesis in skeletal muscle (Li and Wassner, 1986). Accelerated muscle proteolysis is the primary cause of the loss of muscle protein in CKD, while the influence of CKD on protein synthesis is much less prominent than an increase in protein degradation (Mitch and Goldberg, 1996).

Specific complications uncovered in CKD generally occur in other catabolic conditions. These complications which impair IGF-1 signaling to some extent usually include insulin resistance, metabolic acidosis, inflammation, and so on. Kopple et al. (2007) proved that exercise training increases the muscle IGF-I protein level of maintenance hemodialysis patients. Another study indicates that serum IGF-1 and skeletal muscle IGF-1 and IGF-1 mRNA were reduced in CRF rats, elucidating the impaired actions of IGF-1 on protein synthesis and degradation in skeletal muscle of CRF rats (Ding et al., 1996). Skeletal muscle IGF-I promotes skeletal muscle protein synthesis and hypertrophy and suppresses protein degradation (Musarò et al., 2001). In CKD patients, impaired responses to IGF-1 result in suppression of the phosphatidylinositol 3-kinase/Akt (PI3K/Akt) signaling pathway. Then, the p-Akt decreases, leading to reduced phosphorylation of the family of forkhead transcription factors (FoxO1, 3, and 4). When these factors are not phosphorylated, they can translocate to the nucleus to stimulate transcription of the muscle-specific E3 Ub ligases, Atrogin-1 and MuRF-1. The expression of these E3 Ub ligases stimulates muscle protein degradation in the UPS (Lee et al., 2004), which is now widely accepted as the ubiquitin–proteasome pathway resulting in muscle atrophy.

While too many factors can affect the nutritional status of CKD patients, a combination of therapeutic approaches are required to prevent or reverse PEW. These approaches involve optimal nutritional support, correction of acidosis, and physical exercise, which are insufficient to reestablish muscle mass and strength in this vulnerable population. Novel treatment strategies are urgently needed. According to the above mechanisms, related potential pharmacological therapy has been examined. Administration of supraphysiologic doses of anabolic steroids increases muscle size and strength in patients with various CKD conditions (Oliveira et al., 2019). Recombinant human growth hormone (rhGH) has also been examined in maintenance dialysis patients and can improve nutritional biomarkers. Thus, pharmacologic doses of rhGH are expected to be another potential anabolic therapy for maintenance dialysis patients. As SIRT (sirtuins) protein blocks the activities of the transcription factors FoxO1 and FoxO3 (Lee and Goldberg, 2013), SIRT1 activation represents an attractive possible novel pharmacological approach to prevent muscle wasting (Tonkin et al., 2012). In addition, ubiquitin–proteasome inhibitors could be a future treatment option in muscle wasting patients induced by CKD. While too many factors can affect the nutritional status of CKD patients, a combination of therapeutic approaches are required to prevent or reverse PEW. At present, there is no FDA-approved pharmacologic approach to prevent or attenuate wasting in CKD patients. Active research into direct pharmacological treatment based on preclinical translational research and subsequent randomized controlled trials is urgently required.

The last several years have seen the use of TCM as an alternative treatment in patients with CKD in China and other Asian countries (Zhong et al., 2015). The major effects of TCM are related to anti-inflammatory, antioxidative, antifibrotic, and immunomodulatory pathways (Wojcikowski et al., 2006; Zhong et al., 2015). YQJPXYXZ is a traditional Chinese herbal formula and has been used to treat CKD with good efficacy for many years. YQJPXYXZ can improve renal function and clinical symptoms of CKD patients with qi deficiency and blood stasis syndrome. YQJPXYXZ can protect the remnant kidney function and against malnutrition in a 5/6 nephrectomized rat model. To investigate the possible mechanism of the YQJPXYXZ formula, in the present study, we experimented on 5/6 nephrectomy-induced CKD rats. We observed that both KA and YQJPXYXZ formulas had similar effects of delaying muscle atrophy. Results have shown that YQJPXYXZ significantly prevented body weight loss and muscle fiber size decrease and improved protein depletion. Moreover, YQJPXYXZ could increase the IGF-1 level of serum and skeletal muscle in CRF rats, enhance phosphorylation level of Akt, and decrease the Atrogin1 and MuRF1 mRNA and MuRF1 proteins. These results confirmed the potential pharmacological targets underlying and modulating the IGF-1/PI3K/Akt signaling pathway along with inhibiting the UPS of YQJPXYXZ, which exerts its preventive and therapeutic effects in 5/6 nephrectomized rats.

In summary, our data revealed that the YQJPXYXZ formula could delay muscle wasting, which is associated with modulating the IGF-1/PI3K/Akt signaling pathway and inhibiting the UPS. Further efforts are required to improve our understanding of the mechanisms of the YQJPXYXZ formula and perform well-designed RCTs to confirm its efficacy and safety.

## Data Availability

The original contributions presented in the study are included in the article/Supplementary Material; further inquiries can be directed to the corresponding authors.
